# Near-Infrared Imaging of Artificial Enamel Caries Lesions with a Scanning Fiber Endoscope

**DOI:** 10.3390/s19061419

**Published:** 2019-03-22

**Authors:** Robert C. Lee, Yaxuan Zhou, Sara Finkleman, Alireza Sadr, Eric J. Seibel

**Affiliations:** 1 Department of Orthodontics, School of Dentistry, University of Washington, 1959 NE Pacific St, Seattle, WA 98195, USA; csrlee@uw.edu (R.C.L.); sfinkl@uw.edu (S.F.); arsadr@uw.edu (A.S.); 2 Human Photonics Lab, Department of Mechanical Engineering, University of Washington, 4000 Mason St, Seattle, WA 98195, USA; yaxuanzh@uw.edu; 3 Department of Electrical and Computer Engineering, University of Washington, 185 E Stevens Way NE, Seattle, WA 98195, USA; 4 Biomimetics Biomaterials Biophotonics & Technology (B4T) Lab, Department of Restorative Dentistry, School of Dentistry, University of Washington, 1959 NE Pacific St, Seattle, WA 98195, USA

**Keywords:** near-infrared imaging, reflectance imaging, micro computed tomography, dental caries detection, demineralization, quantitative enamel imaging

## Abstract

Several studies have shown that near-infrared imaging has great potential for the detection of dental caries lesions. A miniature scanning fiber endoscope (SFE) operating at near-infrared (NIR) wavelengths was developed and used in this study to test whether the device could be used to discriminate demineralized enamel from sound enamel. Varying depths of artificial enamel caries lesions were prepared on 20 bovine blocks with smooth enamel surfaces. Samples were imaged with a SFE operating in the reflectance mode at 1310-nm and 1460-nm in both wet and dry conditions. The measurements acquired by the SFE operating at 1460-nm show significant difference between the sound and the demineralized enamel. There was a moderate positive correlation between the SFE measurements and micro-CT measurements, and the NIR SFE was able to detect the presence of demineralization with high sensitivity (0.96) and specificity (0.85). This study demonstrates that the NIR SFE can be used to detect early demineralization from sound enamel. In addition, the NIR SFE can differentiate varying severities of demineralization. With its very small form factor and maneuverability, the NIR SFE should allow clinicians to easily image teeth from multiple viewing angles in real-time.

## 1. Introduction

Since the introduction of water fluoridation in the 20th century, the prevalence and severity of dental caries has been constantly decreasing in the United States [1,2]. In addition to water fluoridation, improved oral hygiene and access to dental care, as well as the increased availability of fluoride dentifrices and professional fluoride applications, have all contributed to this shift in caries trends [3,4]. However, dental caries remains a significant problem in the United States [1,5,6]. The majority of newly discovered caries lesions are located in pits and fissures of posterior teeth and on interproximal surfaces between adjacent teeth where they are difficult to detect [7,8,9]. If caries lesions are detected early, they can be treated via non-surgical interventions and loss of tooth structure is avoided [7,8].

Early signs of enamel caries lesions or demineralization are initially seen as “white spot lesions” (WSLs). White spot lesions are increasingly common among children, especially those who go through orthodontic treatment [9]. WSLs have subsurface demineralization with increased porosity, which often cause changes in the optical properties of enamel and can be aesthetically displeasing [9]. WSLs typically form around high plaque stagnation areas such as gingival margins, pits, and interproximal surfaces of teeth, and intraoral appliances such as orthodontic brackets and bands. If caries lesions are detected early, non-surgical treatment options to reverse the caries process such as topical fluoride and calcium phosphate-based remineralization agents can be implemented before the lesion has progressed [10]. Therefore, the timely diagnosis of caries and detection of early demineralization is critical.

Current methods for caries lesion detection and assessment are composed of a combination of visual and tactile exams, which are prone to subjective bias and interference from surface staining. In addition, visual and tactile exams are limited to exposed surfaces, and there is the potential for permanently damaging the intact lesion surface layer from sharp dental instruments [11,12]. Intraoral radiography is most commonly used as an adjunct to visual and tactile examinations for the evaluation of proximal and occlusal caries lesions. Although intraoral radiography is highly specific in detecting advanced caries lesions, its sensitivity is low as intraoral radiography is not capable of showing very early stages of demineralization [13,14]. Moreover, intraoral radiography can be technique-sensitive—overlapping crown structures and radiopaque materials, such as restorative materials and orthodontic appliances, can interfere with imaging, making caries detection difficult. In addition, the risk of exposure to ionizing radiation cannot be underestimated. Therefore, there has been an emphasis on the need for new caries detection techniques that allow for real-time imaging to monitor the progression of early caries lesions [15,16].

Optical diagnostic methods exploit changes in the fluorescence or light scattering of the caries lesion and therefore have great potential for the detection and assessment of caries lesions [17,18]. Near-infrared (NIR) spectrum is the ideal range for high-contrast imaging of dental caries due to increased light scattering to caries affected dental tissue [19]. Previous studies have shown transparency of sound enamel in the NIR wavelength, where the attenuation coefficient is 20 to 30 times lower than that in the visible region, and the enamel has the highest transparency near 1310-nm [20,21]. Recent studies on NIR reflectance imaging with wavelengths coincident with high water absorption, at 1460-nm and beyond 1500-nm, demonstrated high lesion contrast for artificial and natural enamel caries lesions, while also eliminating the influence from stains, which are common in visible-light imaging [22,23].

**Figure 1:**
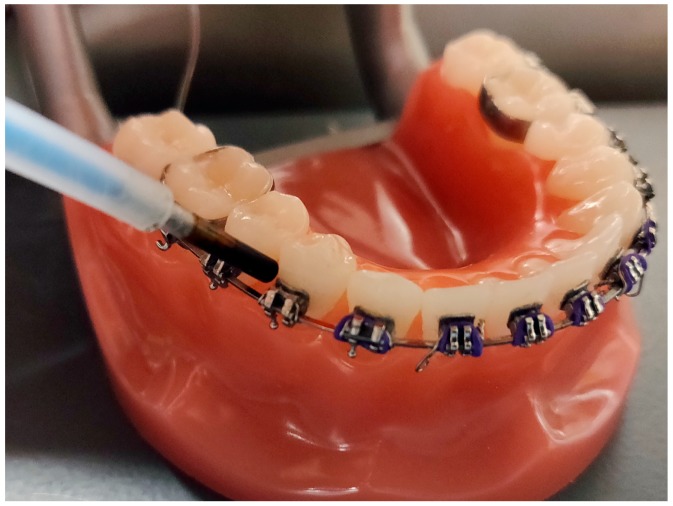
Near-infrared (NIR) scanning fiber endoscope (SFE) prototype on a typodont with orthodontic appliances.

Scanning fiber endoscope (SFE) uses fiber-scanning-based imaging technology to produce high-quality multi-spectral video. Several studies have demonstrated that SFE can be used intraorally to nondestructively image tooth demineralization. Zhang et al. demonstrated that SFE can be used for reflectance and fluorescence imaging of teeth at 405-nm and 532-nm wavelengths [24]. In addition, the SFE using the visible light wavelength was successfully evaluated for its clinical feasibility in a follow-up study [25]. The NIR wavelength has great potential to be employed as a light source for the SFE for detecting caries lesions in a clinical setting [16,26]. We recently developed a SFE operating at 1460-nm and 1310-nm wavelengths in reflectance mode for high-contrast deep artificial lesions, and we demonstrated that the NIR SFE could be used to detect occlusal and interproximal lesions via multi-perspective multi-spectral imaging [27]. The prototype NIR laser-based SFE device should be able to generate high-quality images of shallow artificial lesions with the same magnitude smaller form factor (1.6 mm SFE probe vs. 25 mm diameter NIR camera) at a significantly lower cost. Importantly, the NIR SFE small diameter is the size of a round toothpick and is small enough to fit around interproximal surfaces and intraoral appliances, such as orthodontic brackets, as shown in Figure 1. With the small form factor and maneuverability, the NIR SFE should allow clinicians to easily image teeth from multiple viewing angles in real-time.

The purpose of the study is to test the hypothesis that artificial enamel caries lesions can be discriminated from sound enamel using the NIR SFE reflectance imaging. The current study presents preliminary data on the ability of the NIR SFE device to accurately detect lesions at varying depths. The null hypothesis is that NIR SFE cannot distinguish artificial lesions from sound enamel.

## 2. Materials and Methods

All bovine teeth used in this study were obtained postmortem from discards of animals raised for commercial slaughter. This study did not require the approval of an animal research ethics committee.

### 2.1. Sample Preparation

Based on an effect size of 0.75 derived from a previous study, alpha of 0.05, and power of 0.95, a minimum of 40 samples were needed for this study [28]. Twenty bovine mandibular central incisors were acquired from a slaughterhouse; each tooth was originated from a different cattle. Twenty bovine enamel blocks were prepared 8–12-mm in length with a width of 3 mm and a thickness of 3 mm. The facial surfaces of the bovine enamel blocks were ground to a 9-µm finish. Each bovine enamel sample was partitioned into five windows (1 sound and 4 lesion windows) by creating thin trough lines approximately 1.5 mm apart using a 392-diamond bur (KaVo Kerr, Washington, DC, USA). Total sample size used in this study was 100. Each sample was mounted on a 1 cm × 1 cm × 1 cm black polyoxymethylene block.

Simulated caries lesions were created using artificial demineralization to assess the performance of the new NIR SFE device. The order of the five windows was randomized using a random number generator. A thin layer of acid-resistant varnish in the form of nail polish, Revlon (New York, NY, USA) was applied to the sound window to prevent exposure to the demineralization solution. Samples with four exposed windows were immersed in a demineralization solution maintained at 37 °C at pH 4.9 composed of a 40 mL aliquot of 2.0 mmol/L calcium, 2.0 mmol/L phosphate, and 0.075 mol/L acetate; the surface-softening subsurface lesion model was employed to simulate an active early demineralization in vitro. Acid-resistant varnish was applied to each window after 8, 16, 24, and 32-h of demineralization to protect it from further demineralization. After the 32-h of demineralization, the samples were removed from the demineralization solution, and the acid-resistant varnish was removed by immersion in acetone in an ultrasonic bath for 15 min. Each sample was stored in 0.1% thymol solution to prevent fungal and bacterial growth.

### 2.2. Micro Computed Tomography (µCT), Lesion Depth, and Relative Integrated Mineral Loss (ΔZ)

The µCT (X5000, North Star Imaging, Rogers, MN, USA) images were acquired by a trained engineer at the Computed Tomography Facility at the University of Washington. A previous study has shown that µCT can be used as a non-destructive alternative to transverse microradiography with comparable parameters for the study of enamel lesions [29]. The X-ray source was set at 58 kV, 231 µA, and 13.4 µm focal spot size. The detector panel had pixel pitch of 200 µm × 200 µm. The distance between the X-ray source and the sample was 28.9 mm, the distance between X-ray source and the detector was 454.57 mm, and the zoom factor was 15.7. Each sample was mounted on a custom 3D-printed holder to ensure proper and repeatable positioning for imaging. In total, 720 projections were acquired on each sample in approximately 36 min.

Image reconstruction was initially performed using the default software of the system (efX, North Star Imaging, Rogers, MN, USA). efX is proprietary software that performs automatic calibration for reconstruction process optimization. A physical geometry tool is scanned to avoid common errors with manipulator type of calibration (i.e., geometry issues, system accuracy derivation, measurement uncertainty). This software delivers an optimized µCT resolution and enables data output in a common 3D format such as raw used in this study. Smoothing and ring-artifact correction were done automatically by the efX software for all volumes, and any additional manual filters were not applied. Beam-hardening correction was set at 0.3 in the software.

Data acquired from µCT was used to measure the volume percent mineral content in the demineralization windows of each sample. The µCT scan was transformed in 3 dimensions so that the surface of the sound window was perpendicular to the long-axis of the lesion. The same regions of interest (ROIs) of 700 µm × 700 µm from the NIR SFE images were selected in the µCT scan images and the mean value of each layer was recorded to create a mineral density profile. The mineral density profile was converted to relative mineral density profile by assuming that sound enamel consisting of a maximum mineral density of 86 vol% [30,31].

Lesion depths were estimated by comparing the relative mineral density profile of the lesion window to that of the sound window. From the edge of sound window, the volume percent mineral content over 200 µm depth was recorded. The relative integrated mineral loss (volume percent mineral loss × µm), ΔZ, was calculated by subtracting the volume percent mineral content of each lesion window from that of the sound window.

### 2.3. Near-Infrared Scanning Fiber Endoscope (NIR SFE)

**Figure 2:**
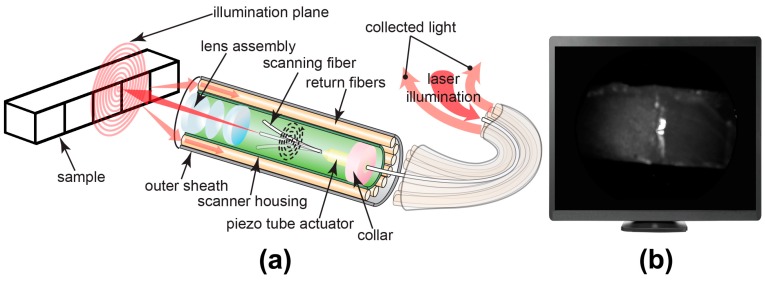
(**a**) The image on the left shows the tip of the NIR SFE imaging the test sample with NIR scanning laser light in a spiral pattern. The rigid length containing the vibrating illumination fiber and lenses is 9-mm long and 1.6-mm in diameter. (**b**) The image on the right shows a NIR SFE digital image displayed on a computer monitor, which matches the field of view shown in (**a**).

As shown in Figure 2, a single-mode fiber cantilever (SM1250G80, Thorlabs Inc., Newton, NJ, USA) and a custom piezo tube actuator were mounted by collar in the center of the probe housing [27]. In each imaging frame, four piezo tube electrodes applied sinusoidal signals with phase difference and increasing amplitudes on the actuator. Then, the actuator drove the central fiber cantilever to scan in an increasing spiral pattern. NIR SFE uses 1310-nm (LPSC-1310-FC, Thorlabs Inc., Newton, NJ, USA) and 1460-nm (QFBGLD-1460-150, Thorlabs Inc., Newton, NJ, USA) lasers as the light source; these two wavelengths were chosen since previous studies have shown that the caries lesions can be imaged with high contrast in reflectance at 1310-nm and 1460-nm [22,32]. The scanning laser beam is focused onto the target by a lens assembly encapsulated in the probe tip. Backscattered light is then collected in a non-confocal manner by ten multi-mode fibers (FPL1009S, Thorlabs Inc., Newton, NJ, USA) mounted around the scanner housing. InGaAs-based near-infrared photodiode (FGA21, Thorlabs Inc., Newton, NJ, USA) is used to measure the intensity of collected backscattering light. As the central scanning fiber scans 250 spirals with increasing deflection amplitude and then is driven back to center by braking signal, a frame is generated by mapping the intensity at each position to its corresponding pixel. The frame rate is currently at 7 Hz and can be increased to 30 Hz for video-rate imaging. The probe has a diameter of 1.6 mm and a rigid length of 9 mm imposed by the piezo tube actuator and lens assembly. With the design of the fiber scanner, the SFE probe is over 800 times smaller in volume than the smallest commercial InGaAs camera (MQ022HG-IM-SM5X5-NIR, XIMEA, Münster, Germany). The probe also provides sufficient image quality with a 60° expandable field of view. Spatial resolution is highest (35 µm) at a working distance of 2 mm and decreases with enlarged image size and greater separation distance.

The probe was mounted at an angle of approximately 30° in order to avoid specular reflection from the sample surface. The probe-to-sample distance was approximately 5 mm. A checkerboard test target imaged prior to imaging samples for calibration. Four sets of images were taken using the NIR SFE—wet at 1310-nm, dry at 1310-nm, wet at 1460-nm, and dry at 1460-nm. Wet samples were removed from 0.1% thymol solution and placed in a sample mount. A folded sheet of Kimwipe^TM^ (Kimberly-Clark, Irving, TX, USA) was placed near the edge of each sample to allow for absorption of the excess water on the surface. The 5 windows on each sample were imaged in separate frames using both 1310-nm and 1460-nm lasers from the left to right window. After imaging the wet samples, the samples were dried with pressurized air for 5 s and the imaging sequence was repeated.

### 2.4. NIR SFE Image Analysis

Images obtained from the NIR SFE were automatically analyzed using a dedicated program constructed with Labview^TM^ (National Instrument, Austin, TX, USA) software. A region of interest (ROI), 20 × 20 pixel (approximately 700 µm × 700 µm), was specified for the sound region of each sample in order to discriminate between demineralized and sound enamel. To minimize variation in angulation of incident light and specular reflection, the same ROI was selected for each window and the mean was recorded. The following measurements were recorded—raw intensity measurement at dry, raw intensity measurement at wet, raw intensity measurement difference between dry and wet conditions, and lesion contrast measurement. The lesion contrast was calculated using (I_L_ − I_S_)/I_L_ [33].

Once the most optimal method for NIR SFE analysis was identified, the measurements of the lesion windows from that specific method were subtracted by the measurement of their corresponding sound windows for the linear regression analysis versus µCT measurements. This relative raw intensity measurement calculation was done to normalize the NIR SFE optical measurement values based on the sound measurements as reference measurements.

The performance of the NIR SFE prototype was also evaluated for the most optimal method. Each window of the sample was ranked in an increasing order of measurements and the lowest ranking window was considered as a sound window. Sensitivity and specificity analysis at each time point of demineralization was performed.

### 2.5. Statistical Analysis

Sample groups were compared using repeated measures one-way analysis of variance (ANOVA) with a Tukey–Kramer post hoc multiple comparison test. Linear regression analyses with robust standard least squares regression and robust generalized estimating equations (GEE) were used to examine the relationship between NIR SFE and µCT measurements. All statistical analyses were performed with 95% confidence with Prism^TM^ (GraphPad software, San Diego, CA, USA) and R 3.5.1 using the GEE package for generalized estimating equations.

## 3. Results

**Figure 3:**
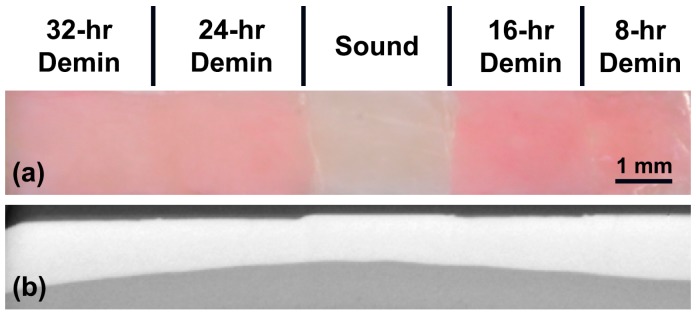
(**a**) Visible light reflectance image and (**b**) the corresponding µCT section image.

**Figure 4:**
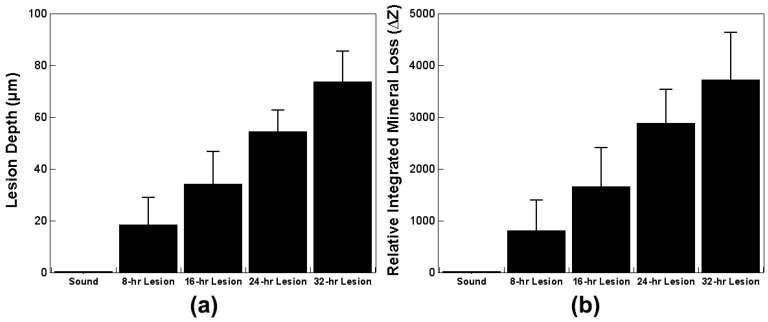
Mean ± S.D. for (**a**) lesion depth and (**b**) ΔZ from µCT measurements.

Figure 3a,b shows the visible reflectance image and the µCT sectional image of a sample, respectively. Surface demineralization is clearly visible in the lesion windows. The sound window can be easily discerned from lesion windows by visual inspection. Lesion windows retained a red hue as it was difficult to completely remove protective red varnish even after immersion in acetone in an ultrasonic bath. To investigate the staining from red varnish affects the NIR SFE measurements further, 10 additional demineralized bovine enamel samples were prepared and imaged before and after the application and removal of red varnish. There was no significant difference in NIR SFE measurements due to the staining from red varnish, and the results can be found in Appendix (Table A1). The samples demonstrate a generalized increase in lesion depth and ΔZ with increased exposure to demineralization solution as shown in Table 1 and Figure 4.

**Figure 5:**
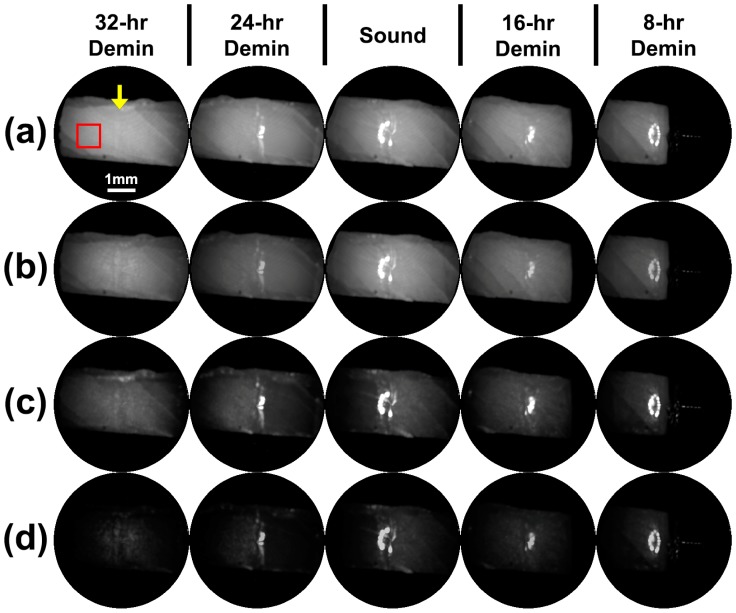
Raw NIR SFE image of the sample shown in Figure 3. Four sets of images were taken: (**a**) dry sample imaged with SFE operating at 1310-nm, (**b**) wet sample imaged with SFE operating at 1310-nm, (**c**) dry sample imaged with SFE operating at 1460-nm, and (**d**) wet sample imaged with SFE operating at 1460-nm. The left window in the SFE field of view is the corresponding window labeled on the top. The red square box outlines the region of interest (ROI) where the mean value was recorded for statistical analysis. The same position of ROI was used for all images of each sample. A yellow arrow indicates the trough line dividing two windows shown in an image frame.

Figure 5 shows raw grayscale reflectance images taken of the dry and wet sample shown in Figure 3 using NIR SFE operating at 1310-nm and 1460-nm. The images were produced in 8-bit or 256 levels of grayscale and the range was not modified. Demineralized dry windows demonstrate a generalized increase in intensity compared to sound window and their wet counterparts. Images taken using 1310-nm light exhibit subsurface anatomical defects such as internal cracks. Images taken using 1460-nm light better visualize surface characteristics such as surface defects or porosity due to demineralization better than images taken using 1310-nm light. Even though the surfaces of the samples were tilted away from the perpendicular angle of the incident to eliminate specular reflection, trough lines between windows exhibited a combination of specular reflection and strong scattering from roughened and angled trough surfaces. The specular reflection from the trough lines was much stronger in the wet sample due to water acting as smooth reflective surfaces.

**Figure 6:**
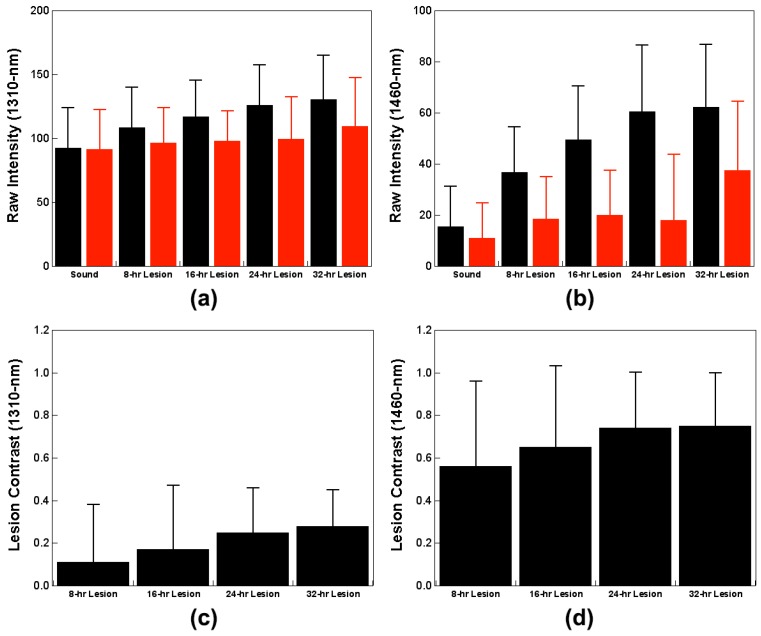
Mean ± S.D. for NIR SFE raw intensity measurements taken using (**a**) 1310-nm and (**b**) 1460-nm light, and lesion contrast measurements taken in the dry condition using (**c**) 1310-nm and (**d**) 1460-nm light. Left (black) bar in each group in raw intensity measurements (**a**,**b**) represents measurements taken when the samples were dry and the right (red) bar in each group represents measurements taken when the samples were wet.

Measurements from NIR SFE imaging of samples in both wet and dry conditions are shown in Table 2 and Figure 6. The raw intensity measurements taken in the dry condition using 1460-nm light demonstrate significant differences between windows with an exception of the 32-h window. The raw intensity measurements in the dry condition increased with an increase in lesion severity in images taken both at 1310-nm and 1460-nm. There was a significant difference between the sound window and the demineralization window for the raw intensity measurements taken in the dry condition using 1460-nm light. However, the raw intensity measurements taken in the dry condition using 1310-nm light did not show a significant difference between the sound window and early demineralization windows, 8-h and 16-h. Raw intensity measurements taken in the wet condition were not significant at both 1310-nm and 1460-nm.

Other measurements were also taken at different conditions to determine the optimal method for differentiating lesion severity. Other measurement methods, such as intensity difference between dry and wet measurements and lesion contrast that have been previously employed in caries detection studies using NIR camera systems, were also compared [28,32]. Intensity difference values both at 1310-nm and 1460-nm increased with an increase in exposure to the demineralization solution. Lesion contrast measured at 1460-nm was far greater than that measured at 1310-nm. It should be noted that there was high variance for intensity difference and lesion contrast measurements.

Once the raw intensity measurements in the dry condition using 1460-nm light were identified as the most optimal method for evaluating this NIR SFE prototype in this study, the measurements of the lesion windows were subtracted by the measurement of their corresponding sound windows for the linear regression analysis versus µCT measurements. The relative raw intensity measurements in the dry condition using 1460-nm light results are shown in Table 3.

**Figure 7:**
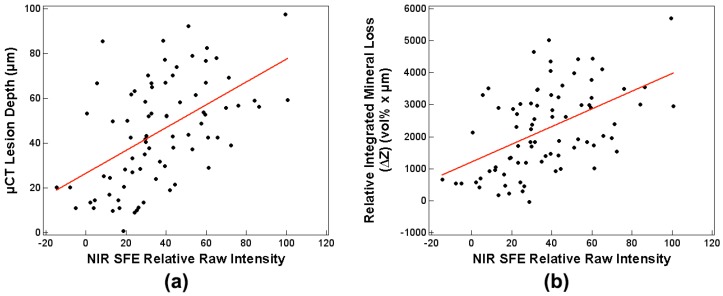
Plots of NIR SFE relative raw intensity measurements in the dry condition using 1460-nm light vs. (**a**) lesion depth and (**b**) relative integrated mineral loss (n = 80).

Sensitivity analyses with robust standard least squares regression between the NIR SFE relative raw intensity measurements taken in the dry condition using 1460-nm light and µCT measurements were performed, and the results are shown in Table 4 and Figure 7. Sensitivity analysis involving robust GEE that accounted for correlation between windows from same sample provided similar results as robust standard least squares regression. In general, the correlation analyses between the NIR SFE raw measurements at dry and lesion depth as well as ΔZ were moderately positive. It should be noted that *p*-values do not account for non-constant variance across different predictor values.

The diagnostic performance of the NIR SFE prototype in the dry condition using 1460-nm light was also evaluated. Each window of the sample was ranked in an increasing order in raw intensity measurements and the lowest ranking window was considered as a sound window. Sensitivity and specificity analyses are reported in Table 5. For all windows, sensitivity was 0.96 and specificity was 0.85. There were three false positives from sound windows and three false negatives originated from early demineralization windows; two false negatives were from the 8-h window and one false negative was from the 16-h window.

## 4. Discussion

This study evaluates the capability of the new NIR SFE device for detection of artificial caries lesions. Previous studies on InGaAs or Germanium CCD-based camera systems operating at near-infrared wavelength have shown promise in detecting and assessing the severity of enamel caries lesions [34,35]. This study demonstrates that the NIR SFE is capable of detecting artificial caries lesions and differentiating between different lesions of varying severities.

The lesion windows of the samples prepared in this study retained a red hue after immersion in acetone in an ultrasonic bath (Figure 3). This is most likely due to increased subsurface porosity from demineralization. The retained red varnish in the lesion windows did not affect the NIR SFE measurements, and the small amount of staining would be comparable to clinical staining of early caries lesions.

Raw intensity measurements taken in the dry condition using 1460-nm light (Table 2 and Figure 6b) showed the most significant differences between windows. Images taken in the dry condition (Figure 5a,c) showed an increase in raw intensity values because the air-filled pores on the surface mineral caused by demineralization act as a scattering medium. Images taken while wet (Figure 5b,d) showed dramatic reduction in raw intensity values as the pores are filled with water negating the strong scatter from the demineralized pores. The images displayed show the raw NIR SFE signals and appear rather dark for 1460-nm images. The visualization of the NIR SFE images can be expected to improve with dynamic range scaling, increasing the power of lasers, and decreasing the distance between sample and probe in a clinical setting.

Other measurement techniques for analyzing NIR images from previous NIR camera studies were also explored in this study to evaluate the best technique for interpreting NIR SFE data [28,32]. The intensity difference measurements (Table 2) between dry and wet measurements and lesion contrast values (Table 2 and Figure 6c,d) showed an increasing trend with an increase in lesion depth and severity; these measurements did not yield as significant results as the raw intensity measurements taken from the dry condition suffered from high variance. Although the lesion contrast measurements (Table 2 and Figure 6c,d) in the dry condition were not significant between lesion windows at both 1310-nm and 1460-nm wavelengths, the reported contrast values at 1460-nm was more than five times higher than those at 1310-nm. Lesion contrast result shows that the images taken at 1460-nm provide superior contrast between sound and demineralized enamel than the images taken at 1310-nm. This indicates that 1460-nm is better suited for detection of demineralization than 1310-nm.

The NIR SFE prototype produced markedly different images taken at 1310-nm and 1460-nm as shown in Figure 5. The images taken at 1460-nm showed much higher contrast images between sound and early demineralization (8 and 16 h) than the images taken at 1310-nm. However, images taken at 1310-nm better depict internal flaws of the samples such as cracks that were most likely introduced during sample preparation because 1310-nm light penetrates enamel much further than 1460-nm light. The difference in the reflectance images captured at these two wavelengths is most likely due to increased water absorption at 1460-nm than 1310-nm [36].

There were moderate positive correlations between the NIR SFE relative raw intensity measurements taken in the dry condition using 1460-nm light and the µCT measurements; Pearson’s r coefficients were 0.52 with the lesion depth and 0.51 with ΔZ (Table 4 and Figure 7). The NIR SFE prototype yielded strong diagnostic performance with high sensitivity (0.96) and specificity (0.85) as shown in Table 5. Variation in depths to underlying dentino-enamel junction and a potential specular reflection from underlying structure may have contributed to the increase in intensity of the false positive sound windows and the decrease in intensity of the false negative lesion windows. However, the three false negatives from early demineralization windows represent very shallow depth of remineralization (18.4 µm mean lesion depth for 8-h window and 34.2 µm mean lesion depth for 16-h window). Detection of early demineralization at such shallow depths is clinically insignificant. It is important to note that there was no false negative in the 24-h and 32-h windows, which represent lesions with greater depths (Table 1 and Figure 4). These results demonstrate that the NIR SFE can be used as a predictor for detecting the presence of early demineralization and for assessing severity of the early lesion. The samples prepared in this study had relatively shallow demineralization, up to 100 µm in depth, as verified by µCT measurements. With more severe lesions than the samples used in this study, future studies may be able to improve the correlation between the NIR SFE measurements and µCT measurements.

The samples used in this study are flat on the surface and the incident light from NIR SFE was intentionally angled to eliminate the specular reflection. The current shortcoming of this NIR SFE prototype is that the specular reflection cannot be eliminated from all angles of view. As the angulation between the surface of the sample and the incident light from NIR SFE vary depending on pixel location in the image, the reflectance raw values of one coordinate in the image to another coordinate are not uniform. There are multiple ways to address this issue. One possibility to overcome this problem is to use cross-polarizers between the laser source and the detector. Another possibility is to use multiple detectors and software optimization for elimination of specular reflection.

Another complication of NIR SFE system is that the measurement is dependent on the distance between the sample and the probe, since the detector in the NIR SFE system collects more photons when the sample is closer to the probe. In this study, all samples and windows were kept at approximately the same distance to reduce this effect, which would have influenced raw intensity values. The sample surface could have been slightly slanted due to errors introduced during sample preparation and mounting on the polyoxymethylene block. At a 5 mm probe-to-sample distance, even a small discrepancy in distance can influence the raw intensity measurements. To overcome this potential distance problem, lesion contrast measurement was also calculated, but the differences among windows were not significant from one another due to high variance. In a clinical setting, if the lesion is small, then imaging both the lesion and surrounding sound enamel in the same field of view will allow an immediate lesion contrast measurement. However, using lesion contrast may present a challenge when the surface of the target is highly curved, such as pits and fissure caries of the occlusal surfaces of the tooth. Multiple images can be taken at different angles to account for the irregular tooth surface morphology. Further study is required for testing NIR SFE for caries detection on non-smooth surfaces of the tooth.

## 5. Conclusions

In summary, the NIR SFE prototype can successfully differentiate between sound and demineralization on smooth enamel surface. The NIR SFE prototype in the dry condition using 1460-nm light was also capable of differentiating varying severity of demineralization. NIR wavelength 1460-nm was more suitable for quantifying superficial caries detection than 1310-nm. Although this study was conducted in a well-controlled manner, there are still necessary improvements to be made to reduce specular reflection and to optimize image quality and calibration. With the advantage of miniature probe size, maneuverability, and real-time imaging speed, NIR SFE has strong potential to be employed in clinical settings for detecting and monitoring early caries lesions, such as WSLs.

## 6. Patents

E.J.S is an inventor of the following two patents on scanning fiber endoscope system, both owned by University of Washington which are licensed to VerAvanti Inc., Redmond, WA, USA:

Seibel, E.J.; Furness, III, T.A. Miniature image acquisition system using a scanning resonant waveguide. U.S. Patent Application No. 6,294,775, 25 September 2001.

Seibel, E.J. Medical imaging, diagnosis, and therapy using a scanning, single optical fiber system. U.S. Patent Application No. 6,975,898, 13 December 2005.

Y.Z. and E.J.S. are co-inventors of the following patent filing on using SFE for optical pH measurement, owned by the University of Washington. Co-inventors participate in a royalty sharing program with the University of Washington.

Seibel, E.J.; Gong, Y.; Xu, Z.; McLean, J.S.; Zhou, Y. System and method for ranking bacterial activity leading to tooth and gum disease. Patent Application No. WO2018081637A1, 3 May 2018.

## Figures and Tables

**Table 1:** Mean ± S.D. for lesion depth and ΔZ from µCT measurements. Groups with the same letters are statistically similar, *p* > 0.05 in each row (n = 100).

	Sound	8-h Lesion	16-h Lesion	24-h Lesion	32-h Lesion
Lesion Depth (µm)	0	18.4 ± 10.6	34.2 ± 12.5	54.4 ± 8.3	73.7 ± 11.8
	a	b	c	d	e
Relative Integrated Mineral Loss, ΔZ (vol% × µm)	0	809 ± 589	1663 ± 745	2687 ± 644	3723 ± 910
	a	b	c	d	e

**Table 2:** Mean ± S.D. for raw intensity, intensity difference between dry and wet images, and lesion contrast values from NIR SFE measurements. Groups with the same letters are statistically similar, *p* > 0.05 in each row (n = 100).

	Sound	8-h Lesion	16-h Lesion	24-h Lesion	32-h Lesion
NIR SFE operating at 1310-nm					
Raw Intensity—Dry	92.7 ± 31.3	108.3 ± 31.5	116.9 ± 28.7	126.2 ± 31.7	130.4 ± 34.3
	a	a,b	a,b,c	b,c	c
Raw Intensity—Wet	91.6 ± 31.2	96.7 ± 27.5	98.1 ± 23.3	99.4 ± 33.1	109.4 ± 38.0
	a	a,b	a,b	a,b	b
Intensity Difference	1.1 ± 7.0	11.7 ± 13.9	18.7 ± 22.6	26.8 ± 21.0	21.0 ± 22.1
	a	b	a,b	c	b
Lesion Contrast ^1,2^ —Dry	-	0.11 ± 0.27	0.17 ± 0.30	0.25 ± 0.21	0.28 ± 0.17
		a	a,b	a,b	b
NIR SFE operating at 1460-nm					
Raw Intensity—Dry	15.5 ± 15.7	36.7 ± 17.8	49.5 ± 20.9	60.6 ± 26.0	62.3 ± 24.5
	a	b	c	d	c,d
Raw Intensity—Wet	11.0 ± 13.8	18.5 ± 16.6	20.0 ± 17.4	18.1 ± 25.7	24.7 ± 27.4
	a	a	a	a	a
Intensity Difference	4.5 ± 5.8	18.3 ± 19.0	29.5 ± 26.9	42.5 ± 26.7	37.6 ± 26.9
	a	b	b,c	c	b,c
Lesion Contrast ^1,2^ —Dry	-	0.56 ± 0.40	0.65 ± 0.38	0.74 ± 0.26	0.75 ± 0.25
		a	a,b	a,b	b

^1^ Lesion contrast values were calculated based on individual raw intensity measurement. ^2^ Number of windows analyzed for lesion contrast was 80 because sound windows were not included (n = 80).

**Table 3:** Mean ± S.D. for raw intensity measurements of lesion windows subtracted by the measurements of their corresponding sound windows from NIR SFE images in the dry condition using 1460-nm light. Groups with the same letters are statistically similar, *p* > 0.05 in each row (n = 80).

	8-h Lesion	16-h Lesion	24-h Lesion	32-h Lesion
Relative Raw Intensity	21.2 ± 17.0	34.0 ± 23.6	45.1 ± 24.3	46.8 ± 22.5
(Lesion-Sound)	a	b	c	b,c

**Table 4:** *p*-value, Pearson’s r, and 95% confidence interval for NIR SFE relative raw intensity measurements in the dry condition using 1460-nm light vs. µCT measurements (n = 80).

	*p*-Value	Pearson’s r (lower CI ^1^, upper CI ^1^)
Lesion Depth	<0.0001	0.52 (0.34, 0.66)
Relative Integrated Mineral Loss, ΔZ	<0.0001	0.51 (0.32, 0.65)

^1^ CI: confidence interval.

**Table 5:** Sensitivity and specificity analysis for NIR SFE measurements in the dry condition using 1460-nm light. A true positive represents a successful detection of demineralization and a true negative represents a successful detection of sound enamel.

	8-h Lesion (n = 20)	16-h Lesion (n = 20)	24-h Lesion (n = 20)	32-h Lesion (n = 20)	All Windows (n = 100)
Sensitivity	0.9	0.95	1	1	0.96
Specificity	-	-	-	-	0.85

## Appendix

**Table A1:** Mean ± S.D., 95% confidence interval, and *p*-value (two-tailed paired t-test) for raw intensity values from NIR SFE measurements of demineralized bovine enamel samples before and after the application and removal of red varnish (n = 10).

	Before	After	*p*-Value	lower CI ^1^, upper CI ^1^
1310-nm	184.2 ± 18.2	184.9 ± 17.0	0.5909	−2.3, 3.8
1460-nm	156.4 ± 41.3	156.0 ± 42.0	0.8942	−7.1, 6.3

^1^ CI: confidence interval.
